# Imaging Tremor Quantification for Neurological Disease Diagnosis

**DOI:** 10.3390/s20226684

**Published:** 2020-11-22

**Authors:** Yuichi Mitsui, Thi Thi Zin, Nobuyuki Ishii, Hitoshi Mochizuki

**Affiliations:** 1 Graduate School of Engineering, University of Miyazaki, Miyazaki 889-2192, Japan; mitsuiyuichi@outlook.jp; 2 Department of Neurology, Faculty of Medicine, University of Miyazaki, Miyazaki 889-2192, Japan; nobuyuki_ishii@med.miyazaki-u.ac.jp (N.I.); mochizuki-h@umin.net (H.M.)

**Keywords:** tremor, essential tremor, ataxia, finger–nose–finger test

## Abstract

In this paper, we introduce a simple method based on image analysis and deep learning that can be used in the objective assessment and measurement of tremors. A tremor is a neurological disorder that causes involuntary and rhythmic movements in a human body part or parts. There are many types of tremors, depending on their amplitude and frequency type. Appropriate treatment is only possible when there is an accurate diagnosis. Thus, a need exists for a technique to analyze tremors. In this paper, we propose a hybrid approach using imaging technology and machine learning techniques for quantification and extraction of the parameters associated with tremors. These extracted parameters are used to classify the tremor for subsequent identification of the disease. In particular, we focus on essential tremor and cerebellar disorders by monitoring the finger–nose–finger test. First of all, test results obtained from both patients and healthy individuals are analyzed using image processing techniques. Next, data were grouped in order to determine classes of typical responses. A machine learning method using a support vector machine is used to perform an unsupervised clustering. Experimental results showed the highest internal evaluation for distribution into three clusters, which could be used to differentiate the responses of healthy subjects, patients with essential tremor and patients with cerebellar disorders.

## 1. Introduction

A tremor is one of the most common involuntary movements seen in neurological disorders. It is characterized as a rhythmic, involuntary oscillation of a body part by muscle innervations that imply repetitive contractions [1,2,3,4]. Various types of tremors occur, depending on their causes. In general, tremors can be divided into two types: resting and action tremors. Action tremors can be further classified into postural tremors, kinetic tremors, task-specific tremors and intention tremors [5,6]. In clinical practice, a tremor is most commonly classified by its appearance and cause or origin. There are actually more than 20 types of tremors. Among them, the most common cause of resting tremors is Parkinson’s disease (*PD*). The most common causes of postural and kinetic tremors are essential tremors (*ET*) and cerebellar disorders (*CD*). It is easy to distinguish *PD* resting tremors from other tremors because trembles occur at rest and weaken when the target muscles contract [4,5,7]. On the other hand, there are various causes of action tremor, and it is not easy to identify the cause.

The most common causes of action tremors are essential tremors (*ET*) and cerebellar disorders (*CD*). *ET* tremor behaves regularly, but *CD* tremor behaves irregularly and sometimes includes intention tremor [5,6]. Both *ET* and *CD* patients have several common features, such as increased tremor when mentally stressed and restricted fine movements. In clinical practice, clinicians try to distinguish these two tremors, *ET* and *CD*, by neurological examinations such as FNF (finger–nose–finger) test [1,6,7]. However, it is not easy to detect subtle irregularities of finger movement and observe where the finger tremor becomes stronger during the FNF test by usual observation. For this reason, distinguishing between *ET* and *CD* could be difficult even for a skilled neurologist. Therefore, we propose a non-contact method of distinguishing *ET* from *CD* featuring image processing technology and including measurement of tremor severity to confirm its effectiveness.

The rest of this paper is organized as follows: In Section 2, we review the literature relating to our research. In Section 3, the materials and methods are proposed. The method of disease diagnosis and analysis is described in Section 4. Then some experimental results are described in Section 5 using datasets collected by two neurologists who specialize in patients with tremors. In Section 6, we present discussions and plans for future research. Finally, we conclude the paper by giving remarks in Section 7.

## 2. Related Works

*ET* is a disease in which tremors only appear as a symptom and are not life-threatening. However, it is clinically important to make an early diagnosis, allowing treatment specific to *ET* when available and improving the patient’s quality of life [8]. The prevalence of *ET* is approximately 2.5–10% of the population [9]. Although it can develop in any age group, it is mainly seen in the elderly: 4% of people over 40 years old and 5–14% of people over 65 years old have *ET* [10,11,12]. Although the cause of *ET* is not well understood, speculation exists that a hyperexcited state of the sympathetic nerve is involved because the symptoms increase with stress [13]. *CD* results from causes such as cerebellar infarction, inflammation, demyelination, autoimmunity, trauma, degeneration and tumors. Symptoms of *CD* include cerebellar ataxia, intention tremor and cerebellar sway of the upper limbs [5,7,12]. *ET* is rarely life-threatening, while *CD* can be. However, *ET* often disturbs a patient’s quality of life. Therefore, an early diagnosis, differentiating *ET* from *CD,* is important. In addition, even in those who are highly skilled, it may be difficult to differentiate tremors when the patient first presents with the symptom.

Currently, the diagnosis of a patient’s disease using the characteristics of tremor is performed subjectively based on the experience and skills of specialists. However, there are various problems with this. Doctors who are not specialists, such as family doctors and on-duty doctors, do not have a means of quantitatively evaluating a tremor, incurring the risk of misdiagnosis. Such quantification is important in determining the proper treatment.

Various tremor rating scales have been used to evaluate symptoms [14,15,16], but these are qualitative and subjective, and errors may occur depending on the person who assesses them [17,18]. Therefore, how to more accurately quantify tremor characteristics has become an urgent subject of research. For example, such research has included the acquisition of tremor signals using devices such as multipolar *EMGs*, electromagnetic tracking devices, accelerometers and gyroscopes, using the resulting data for evaluation and diagnosis [19]. However, since these methods require a large-scale dedicated device, it is unrealistic to use them in an examination room. In addition, these methods often require attaching a sensor or the like to the patient, resulting in different symptoms due to stress or the burden imposed during the examination. Recent tremor-related research has been done using magnetic resonance imaging (MRI) and a neurophysiological assessment [20]. Results indicate a significant association between severe tremors and malfunctions in specific areas of the brain. Moreover, the literature includes applicable developments in image processing in the framework of deep learning [21,22].

The severity assessment of *ET* or *CD* is determined by expert opinion and is likely to be subjective in nature. Several investigators have tried to quantify these symptoms. Analysis of FNF test, a classic neurological examination method, has been reported using an accelerometer or inertial sensor. Using inertial sensors, the changes of spatiotemporal parameters are related to the disability level in patients with multiple sclerosis [23] or cerebellar ataxia [24]. Using a three-dimensional motion capture system, analyses of body movements during FNF test in patients with poststroke could discriminate between patients with mild and moderate upper limb impairments [25]. These studies have been successful in quantifying the severity of symptoms. However, no research has ever tried to capture and distinguish between the characteristics of *ET* and *CD*. Furthermore, the fine movements of the fingertips during the FNF test has never been analyzed by monitoring them with a video over time.

## 3. Materials and Proposed Imaging Method for Tremor Quantification

In this section, we describe the architecture of our proposed system in which tremors are characterized based on visual data collected by performing the *FNF* (finger–nose–finger) test. Specifically, these visual data are collected using a smartphone, as in the actual diagnostic, for distinguishing *ET* from *CD*. The purpose of this analysis is to automatically and objectively diagnose the disease and measure its severity. In the *FNF* test, patients move their index finger back and forth between their nose and the examiner’s finger to see whether tremors occur. As a result, non-specialist doctors such as family doctors or on-duty doctors can avoid misdiagnosis, detecting life-threatening problems, and averting *MRI* imaging and other unnecessary medical costs. In addition, without the need for sensors, no burden is placed on the patient, and the *FNF* test analysis can be performed easily. The system is composed of the following four components: the dataset collection system, image preprocessing, feature extraction, disease diagnosis and analysis.

### 3.1. Subjects and Design of Data Collection System

**Figure 1:**
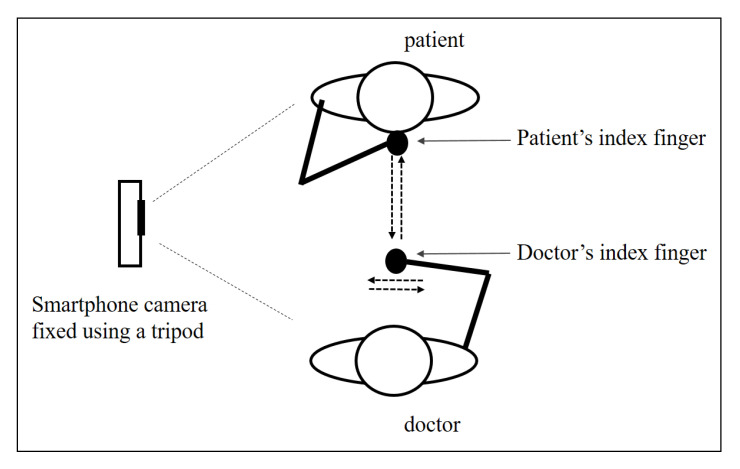
The illustration of the data collection system.

Data collection for the *FNF* test was conducted in an examination room at the Miyazaki University Hospital for *ET* patients (*N* = 10; female, *n* = 4; age, 71.5 +/− 8.1, mean +/− *SD*) and *CD* patients (*N* = 18; female, *n* = 9; age, 68.1 +/− 7.5), with images captured in a side view. Figure 1 illustrates the process of the data collection system. The smartphone camera is fixed at a distance of about 1~1.5 m from the doctor and the patient. The recorded video has a resolution of 480 × 640 pixels, and the frame rate is 30 frames per second (fps). The doctor places his index finger at various locations in front of the patient. The patient touches his/her index finger to the doctor’s index finger and then touches his/her index finger to his/her own nose. Repeat several times with the doctors moving the target finger each time.

Two neurologists made diagnoses and evaluated the severity of the patients’ tremors based on the essential tremor rating assessment scale [15] or the scale for assessing and rating ataxia [14]. According to these scales, patients were classified into two groups; mild (*ET*, upper limb tremor < 1 cm; *CD*, *FNF* test tremor < 2 cm) or severe (*ET*, upper limb tremor ≥ 1 cm; *CD*, *FNF* test tremor ≥ 2 cm). The video data were recorded by two neurologists using smartphones. The data or healthy subjects (*N* = 8; female, *n* = *X*; age, *X* +/− *X*) include data recorded with the cooperation of members of the laboratory, featuring a recorded video of the diagnostic test performed on two ordinary healthy people at the Miyazaki University Hospital. The *FNF* test was performed by winding red or green tape around the subject’s finger. This protocol was approved by the Ethics Committee of the University of Miyazaki, with a waiver of written informed consent obtained from all participants.

### 3.2. Image Preprocessing Component

**Figure 2:**
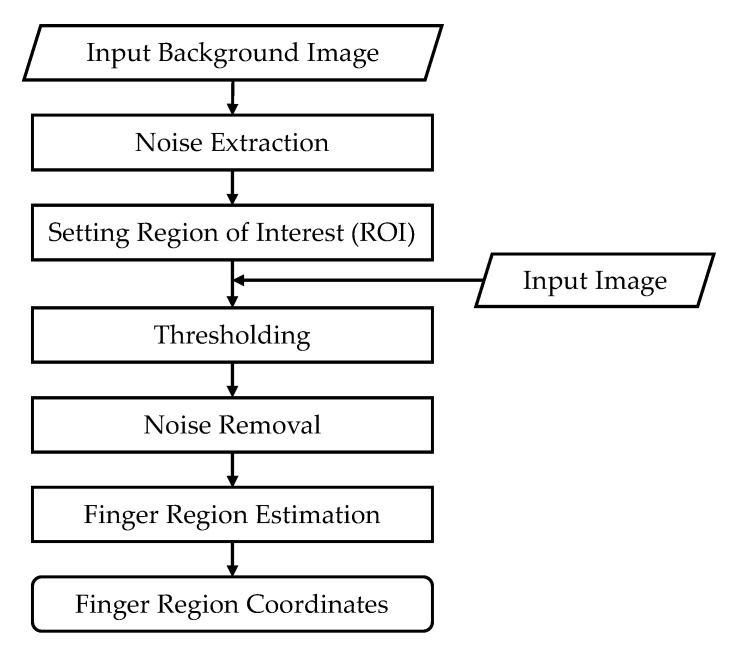
Finger area extraction algorithm.

In this component, image preprocessing is performed in preparation for further analysis. In the first step, the patient’s finger area must be extracted from the video image in each frame. Figure 2 shows the algorithm for extracting the finger area. First, a background image is detected as a noise source, and then the region of interest is set by removing the noise. Subsequent processes include inputting an image for extracting the finger area, threshold processing, noise processing and finger area estimation, finally obtaining the coordinates of the finger area.

**Figure 3:**
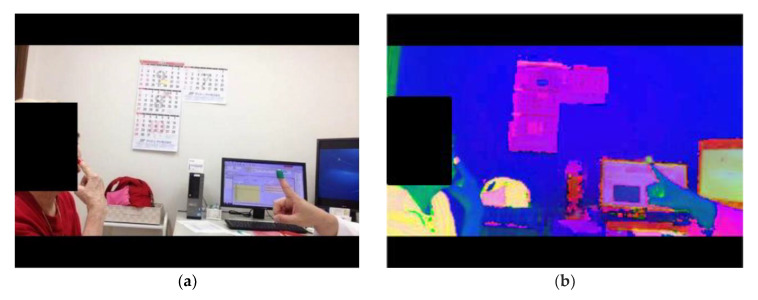
(**a**) Input image. (**b**) The converted HSV image for the input image.

Due to the location for recording video in the examination room, noise can occur for many reasons when extracting the finger area. In the presence of noises, we perform a noise removal process by using background modeling with an initial background as an image that does not include the target object. By converting the RGB image to HSV, we perform the defined thresholding of the hue information in order to obtain the finger object. The input image and converted HSV image are shown in Figure 3a,b, respectively [16]. Hue information representing the hue of the HSV image, Saturation information representing the saturation, and Value information representing the brightness are thresholded, and the finger is obtained by taking the logical product with the region of interest.

We also performed noise processing by calculating the aspect ratio of each area resulting from the labeling process. Since the finger area has a shape close to that of a square, threshold values are applied to the calculated aspect ratio to remove areas of the same size as finger areas that are elongated in vertical or horizontal orientations and could not be removed by noise processing using labels.

#### 3.2.1. Estimation of Finger Area when Multiple Labels Exist

Even if noise processing is performed, not all noise can be removed. In addition, noise processing sometimes removes the finger area. In such a case, the finger area is estimated as follows: If multiple labels exist, such as frame *t*, select the label closest to the coordinates of the finger area surrounded by the red circle detected in the previous frame. The coordinates of the finger area are obtained by calculating the center of gravity of the labeled object.

#### 3.2.2. Estimation of Finger Area when No Label Remains

**Figure 4:**
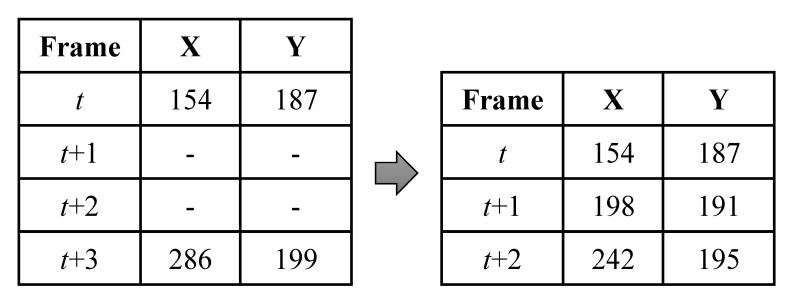
Estimation of finger area when no label remains.

If the finger area is mistakenly removed during noise processing, all labels can be lost. In that case, a smoothing process is performed using finger area coordinates from preceding and subsequent frames to estimate the finger area. For example, as shown in Figure 4, when two frames with no label continue for two successive frames, the difference between *X* coordinate and *Y* coordinates is calculated from the preceding and subsequent frames, and the coordinate values are evenly calculated for the unlabeled frames. The finger area is estimated by substituting a value that changes.

### 3.3. Feature Extraction Process

Now, we present the feature extraction process for the detected finger areas in an *FNF* test. In this process, we extract the following six measures.

#### 3.3.1. Mean Square Deviation (RMSD: Root-Mean-Square Deviation)

**Figure 5:**
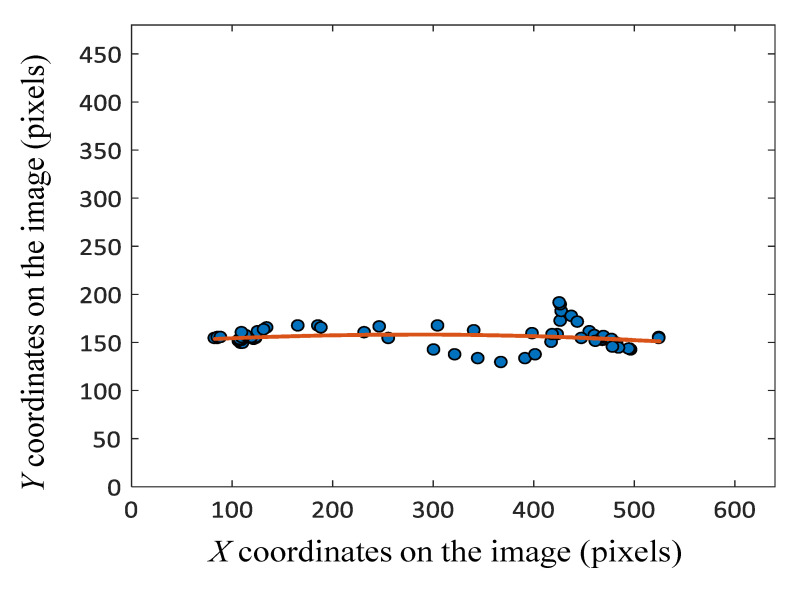
Plot of finger trajectory and approximate curve.

This measure quantifies the vertical distance of the patient’s up and down positions. In Figure 5, the finger region is shown as a graph in the rectangular coordinate plane. In order to do so, we employ a linear-quadratic function along with the least square method. Since the linear-quadratic function represents a parabola curve, we can estimate the vertical distance that the finger moves up and down by computing the root mean square deviation (RMSD) measure. The formula for calculating RMSD is shown below:

(1) $$RMSD=\sqrt{\frac{\sum_{i=1}^{n}{\left(y_{i}-{\overset{⌢}{y}}_{i}\right)}^{2}}{n}},$$

where *n* is the number of plotted data points,$y_{i}$ is the plotted value, and ${\overset{⌢}{y}}_{i}$ is the value of the approximated quadratic function.

#### 3.3.2. Dispersion of Acceleration

As the second measure in consideration, dispersion of acceleration digitizes variations in speed. This measure was selected because healthy people have constant finger movements. However, patients with tremor symptoms have varying rates of change. Therefore, acceleration is calculated for each frame using the coordinate data of the finger region. Next, by calculating the variance from the calculated acceleration, we can quantify the dispersion of acceleration. The formula for calculating this variance is shown below.

(2) $$\mathrm{Variance}=\frac{1}{n}\sum_{i=1}^{n}{\left(x_{i}-\bar{x}\right)}^{2},$$

where *n* is the number of frames, $x_{i}$ is the acceleration in each frame, and $\bar{x}$ is the average value of the acceleration. Using the dispersion of acceleration that digitizes variation in speed change, we can quantify how the patient’s finger is decelerating near the examiner’s finger.

#### 3.3.3. Histogram Feature

In the case of tremor patients, particularly *ET* patients, their fingers often slow down near the examiner’s finger when performing the *FNF* test. Therefore, the following equation is used to determine whether the finger is moving back and forth with a constant rhythm.

(3) $$\mathrm{Histogram}=\frac{\left(h_{max}-h_{med}\right)}{n},$$

where *n* is the number of frames, *h_max_* the maximum value of the histogram, and *h_med_* is the median value of the histogram. The difference between the simple maximum value and the median value requires a different round-trip time (number of frames) depending on the moving image, so normalization is performed by dividing by the number of round-trip frames. In the histogram method, we first construct a histogram by taking the X coordinate on the image of the finger as the vertical axis and the frame number as the horizontal axis. We then divide the range between the maximum frequency of X coordinate and the minimum frequency of X coordinate into four equal parts [12]. Finger movement can be considered unstable if angle analysis indicates that the finger moves up and down an excessive number of times. Moreover, the ratio between the time required in the initial movement of the patient’s finger from the examiner’s finger to the patient’s nose and the time required for the return trip is longer for tremor patients. In this case, the average moving distance is used for digitizing how much the finger is shaking.

#### 3.3.4. Angular Feature

In the *FNF* test, the fingers are moved horizontally, but in patients with tremor symptoms, fine up and down vibrations occur. In order to detect this, the angle at which the finger has moved between frames is considered and calculated by using the following equation.

(4) $$\theta =\tan^{-1}\frac{y_{n}-y_{n-1}}{x_{n}-x_{n-1}},$$

**Figure 6:**
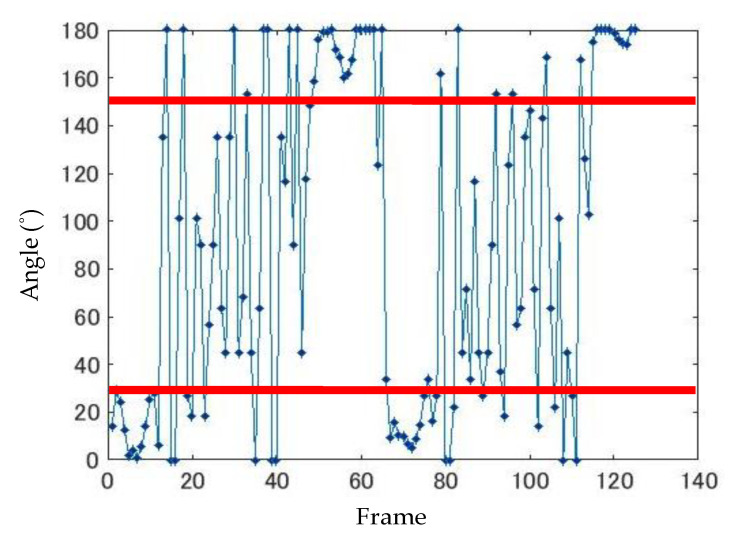
Angle of finger movement.

However, the range of *θ* is −180° ≤ *θ* ≤ 180°. Here, (*x_n_*, *y_n_*) is the coordinate data for the finger region of the *nth* frame, and (*x*_(*n*−1)_, *y*_(*n*−1)_) is the coordinate data for the *n* − 1 frame. The total number of frames satisfying −150° < *θ* < −30° or 30° < *θ* < 150° is determined using the angle obtained from the above equation; the number of times the finger swings up and down is also obtained. An example of the finger movement angle is shown in Figure 6.

#### 3.3.5. Measure of Round-Trip Time Ratio

**Figure 7:**
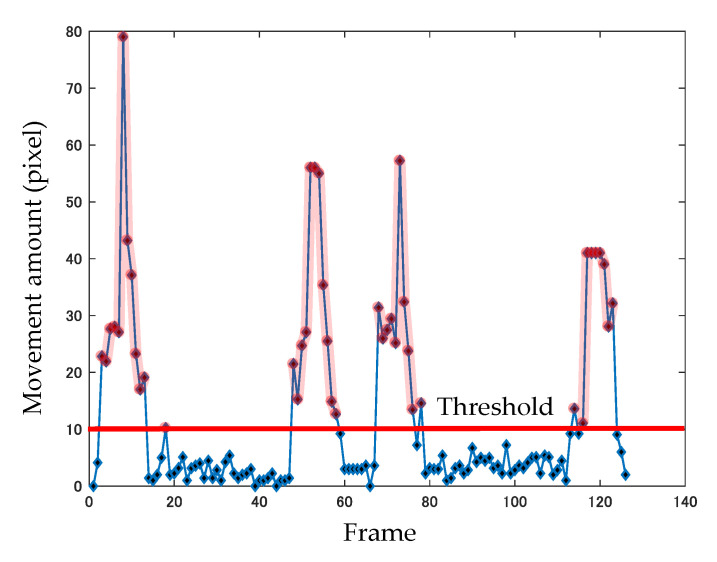
Amount of finger movement in each frame.

In order to detect abnormality in the *FNF* test, the following equation is used to determine the ratio between the time required for initial and return paths in finger movement:

(5) $$\mathrm{Time}\;\mathrm{Ratio}=\frac{{\bar{f}}_{0}}{{\bar{f}}_{r}},$$

where ${\bar{f}}_{0}$ is the average number of frames on the initial path and ${\bar{f}}_{r}$ is the average number of frames on the return path. Here, a threshold value is set for the amount of finger movement in each frame, as shown in Figure 7. The frames in which finger movement exceeds the threshold value are extracted, and the average number of frames for the initial and the return path is obtained. Then, the ratio between the time required for initial and return trips is calculated.

#### 3.3.6. Measure of Average Travel Distance

The final feature to be extracted is the average moving distance digitizing how much the finger is shaking when first touching the examiner’s finger. Tremor patients shake their fingers when touching the examiner’s finger in the *FNF* test. This is especially remarkable in *ET* patients. Therefore, initial and return frames are extracted during the calculation process for the round-trip time ratio. Thus, the average moving distance of the fingers during that period is calculated using the following formula.

(6) $$\bar{d}=\frac{1}{(m_{2}-m_{1})+(m_{4}-m_{3})+2}\left(\sum_{i=m_{1}}^{m_{2}}d_{i}+\sum_{i=m_{3}}^{m_{4}}d_{i}\right)$$

where $\bar{d}$ represents the average moving distance, *d_i_* is the moving distance in the *i* frame, *m*_1_ is the first frame of the first touch, *m*_2_ is the last frame of the first touch, and *m*_3_ is the first frame of the second touch, *m*_4_ is the frame at the end of the second touch.

## 4. Method of Disease Diagnosis and Analysis

A classifier is learned by using the feature values obtained in Section 3, and the disease is diagnosed by classifying the data using that classifier. In this study, we classify by supervised learning. Supervised learning is a method of learning a classifier that correctly outputs the relationship between data and class, using the information on the label for the data and the class of data provided in advance. The methods used as classifiers are as follows: linear discriminant analysis, logistic regression analysis, support vector machine (*SVM*), and the *k*-nearest neighbor method (*k*-*NN* method). Verification of the classifier is performed by *k*-fold cross-validation. Briefly, we will describe these classifiers as follows:

### 4.1. Linear Discriminant Analysis

Linear discriminant analysis is a method of finding a straight line that can best classify which group to enter when new data are obtained using data provided in advance that was divided into different groups.

### 4.2. Logistic Regression Analysis

In medical statistics, logistic regression analysis is one of the statistical methods used in multivariate analysis. In this method, when the objective variable (class) is binary, the probability *P* that an event occurs when one of the classes is an event is expressed by the equations in (7).

(7) $$\begin{array}{l} P^{\prime }=\ln\left(\frac{P}{1-P}\right)=b_{0}+b_{1}x_{1}+b_{2}x_{2}+\ldots +b_{p}x_{p},\\ P=\frac{1}{1-e^{-P^{\prime }}}, \end{array}$$

where *b*_0_ is a constant, *b_p_* is a partial regression coefficient, and *x_p_* is a covariate (feature amount).

### 4.3. Severity Measurement in ET Patients

**Figure 8:**
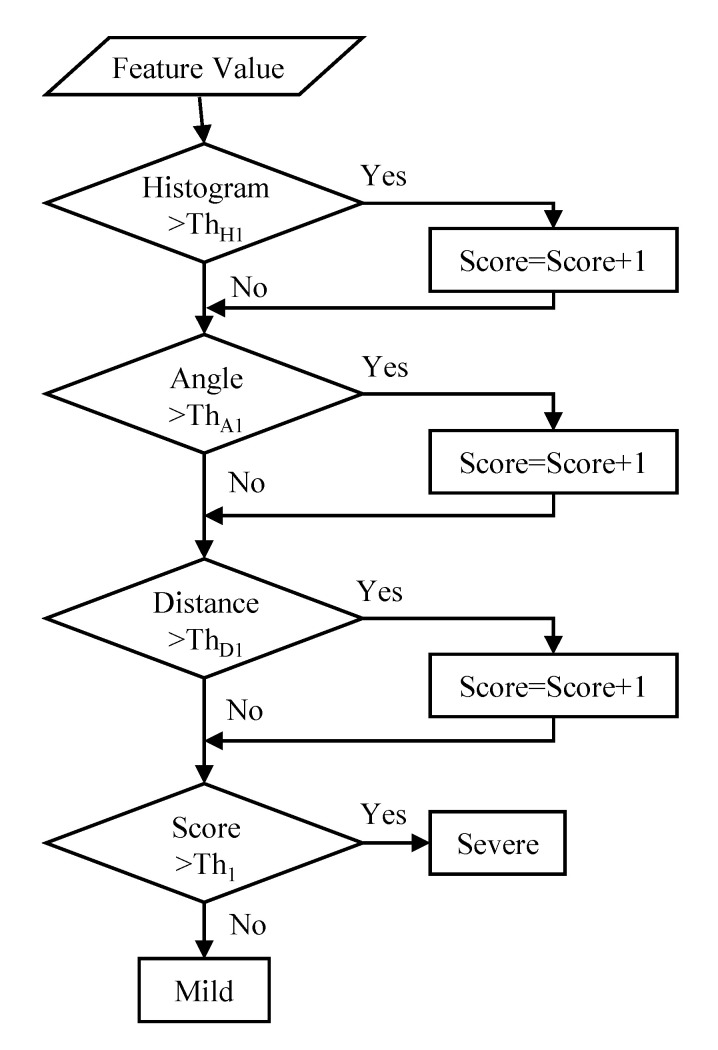
Severity measurement algorithm for essential tremors (*ET*) patients.

Figure 8 shows the algorithm for measuring severity in *ET* patients. Threshold processing is applied to the feature amount calculated by the histogram analysis, angle analysis, and the average moving distance proposed in Section 2, and if it is equal to or greater than the threshold, one point is added to each. If the total number of points finally scored is less than 2, the score is mild, and if the total score is 2 or more, the score is severe.

### 4.4. Severity Measurement in CD Patients

**Figure 9:**
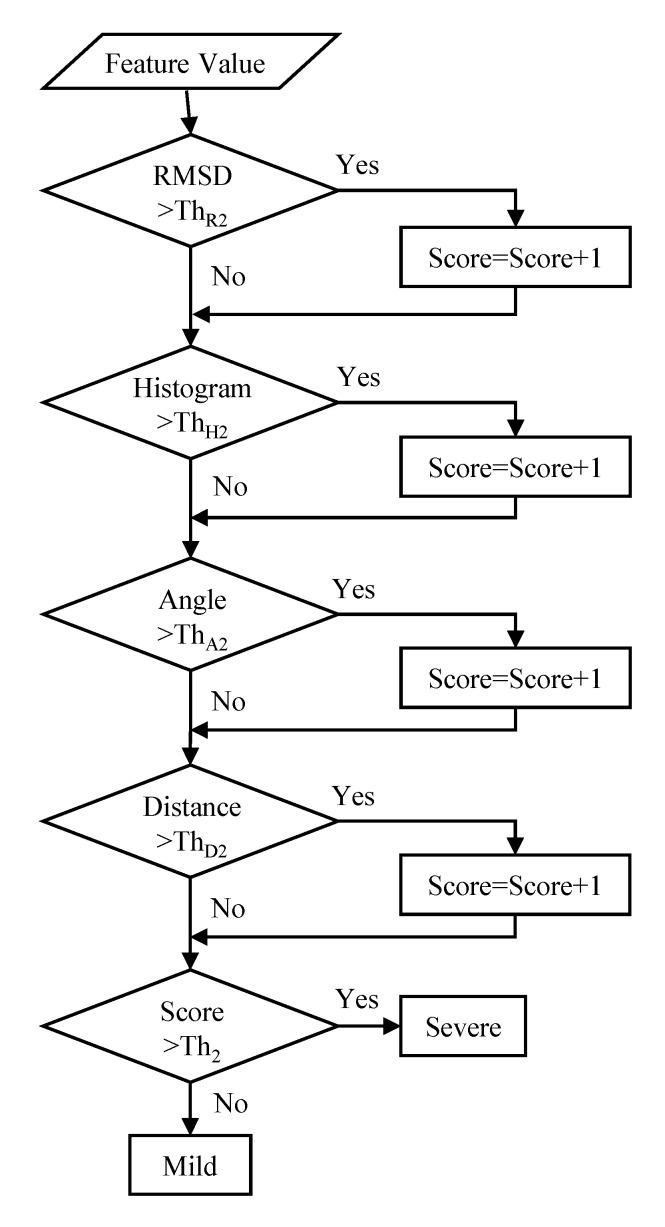
Algorithm for measuring severity in cerebellar disorders (*CD*) patients.

Figure 9 shows the algorithm for measuring severity in *CD* patients. Threshold processing is applied to the feature amount calculated by *RMSD*, histogram analysis, angle analysis, and average moving distance proposed in Section 2, and if it is above the threshold, one point is added to each. If the total score is less than 3 points, the severity score is mild, and if the total score is 3 points or more, the score is severe.

## 5. Experimental Results

This section describes the results of experiments using the proposed method. This time, we conducted an experiment using 8 sets of data for healthy people, 10 data for *ET* patients, and 18 data for *CD* patients. For training and testing data separation, we applied the *k*-fold cross-validation technique. In our system, we set *k* = 5 and therefore, the dataset is split into five folds. Machine learning is performed in experiments that each have multiple classes, as follows: (1) healthy subjects and tremor patients, (2) *ET* patients and *CD* patients and (3) healthy subjects, *ET* patients and *CD* patients. After learning is completed, *k*-fold cross-validation is performed. The results are shown in Table 1, Table 2 and Table 3, respectively.

We have also conducted experiments measuring the tremor severity of *ET* and *CD* patients using the method proposed in Section 3. In these experiments, the threshold was determined by using a total of four training data in experiments featuring examinations by Doctor A and Doctor B, which had a low rate of accuracy in determining the severity of *ET* and *CD* patients. Table 4 and Table 5 provide samples of the experiment results.

## 6. Discussion

As a result of training the classifier using the proposed feature quantity and *k*-division cross-validation, in the classification experiment featuring healthy subjects and tremor patients, Table 1 shows the best results using *SVM*, with an accuracy of 86.7%. In all three misdiagnoses, the examining doctors incorrectly assessed the symptoms to be mild and had difficulty in correctly assessing the symptoms even when reviewing the videos.

According to the classification of *ET* and *CD* patients, from Table 2, the best result was obtained with *SVM*, and its accuracy was 83.6%. Four misdiagnoses occurred, in three of which physicians incorrectly assessed the symptoms to be mild. The data for the single remaining *ET* patient presented *CD*-like characteristics, such as a high *RMSD* value due to severe symptoms with much shaking of the finger up and down and much time for the initial trip from examiner’s finger to patient’s nose. These severe symptoms seem to be the cause of the misclassification.

As shown in Table 3, a classification experiment featuring healthy subjects, *ET* patients and *CD* patients, the best result was obtained using *SVM*, with an accuracy of 76.1%. The accuracy was slightly less than with the above two classification experiments. In order to improve the accuracy, new features that better differentiate classes must be studied, and the dataset should be enlarged.

### 6.1. About Severity Measurement

Improving the measurement of severity first involves adapting the threshold to the amount of feature data, calculating the score, and then taking the measurement. As a result of doing so, the measurement accuracy for both *ET* and *CD* patients could be improved to 75.0%. In one example, the inaccuracy resulted from repeating training for the *FNF* test. In this case, the score was low, and the doctor incorrectly assessed the symptoms as severe, though the experimental results indicate that the symptoms were actually mild. In addition, since the purpose of this study is to facilitate measurements in the examination room, the conditions for recording video, such as camera placement, have not yet been optimized. For this reason, some erroneous results were obtained because the feature amount for the average moving distance of the finger increases when the camera is close and decreases when the camera is far. In order to solve this problem, normalization processing could be added so that the feature amount does not change depending on the conditions of video recording.

### 6.2. Future Outlook

In the future, the main issues will be the examination of new features and the use of new methods. The *ET* tremor is regular, and its frequency is generally 4–12 Hz [4,26,27]. In order to focus on the frequency component of tremors in future research, it is expected that diagnostic accuracy will be improved by performing analysis using the fast-Fourier transform.

Due to the fact that severity is difficult to define, we only differentiated mild and severe symptoms rather than attempting a more granular assessment. As a future challenge, we will quantify the degree of severity. Doing so will allow understanding the effect of therapy and enables doctors to modify prescriptions when symptoms do not improve.

## 7. Conclusions

In this paper, we proposed a non-contact method of discriminating *ET* and *CD* and a method of measuring tremor severity by analyzing the *FNF* test using image processing technology. We proposed feature quantities to quantify what a doctor actually focuses on in the *FNF* test and trained a classifier using these feature quantities. As a result of performing *k*-fold cross-validation on the classifier, *SVM* obtained an accuracy of 83.6% in classifying *ET* and *CD* patients. In addition, threshold processing was applied to the amount of feature data in each dataset, the score was calculated, and the severity was evaluated. As a result, the severity of symptoms for both *ET* and *CD* patients could be evaluated with an accuracy of 75.0%.

In the future, we expect to improve diagnostic accuracy by examining new features and using new methods, including some analysis of frames per second (fps) increase and Eigen background models focusing on tremor frequency and on detecting the nose of the patient and the finger of the examiner. Since the Eigen background model is based on the method of principal component analysis, we expect that a more clear foreground image (in our case, the finger area) would be extracted. It is also necessary to consider various approaches to quantify severity. In addition, we will increase the amount of data collected and aim to build a more reliable system. Moreover, in our future work, we would like to explore and analyze the raw recorded data by using a machine learning approach, such as using recurrent convolutional neural networks to extract prominent features from the data.

## Figures and Tables

**Table 1:** Classification results of healthy subjects and tremor patients.

Classifier	Accuracy (%)
Linear discriminant	83.9
Logistic regression	85.0
*SVM*	86.7
*k*-*NN*	83.4

**Table 2:** Classification results of *ET* patients and *CD* patients.

Classifier	Accuracy (%)
Linear discrimination	79.3
Logistic regression	72.2
*SVM*	83.6
*k-NN*	70.0

**Table 3:** Classification accuracy of healthy subjects, *ET* patients and *CD* patients.

Classifier	Accuracy (%)
Linear discrimination	68.9
*SVM*	76.1
*k-NN*	60.0

**Table 4:** Accuracy of severity measurement in *ET* patients.

Severity Measurement	Total Number	Correct Number	Accuracy (%)
Mild	4	3	75.0%
Severe	4	3	75.0%
Total	8	6	75.0%

**Table 5:** Accuracy of severity measurement in *CD* patients.

Severity Measurement	Total Number	Correct Number	Accuracy (%)
Mild	7	6	85.7%
Severe	9	6	66.7%
Total	16	12	75.0%
